# An Exploration of Black Men’s Attitudes and Experiences Communicating with Dentists about Oral and Pharyngeal Cancer

**DOI:** 10.3390/ijerph20196859

**Published:** 2023-09-28

**Authors:** Patrick D. Smith, Darien J. Weatherspoon, Tiosha Bailey, Caryn E. Peterson, Marcus Murray, Osei Bekoe, Anuoluwapo Shadamoro, Nosayaba Osazuwa-Peters, Kimberly Nu-Tall

**Affiliations:** 1 College of Dentistry, University of Illinois Chicago, 801 S. Paulina St., Chicago, IL 60612, USA; ashada2@uic.edu; 2 School of Dentistry, University of Maryland, 650 W. Baltimore St., Baltimore, MD 21201, USA; dweatherspoon@umaryland.edu; 3 School of Public Health, University of Illinois Chicago, 1603 W. Taylor St., Chicago, IL 60612, USA; tiosha@tbaileyconsultingfirm.com (T.B.); cpeter1@uic.edu (C.E.P.); 4 Project Brotherhood, 1510 E. 55th Street, P.O. 15282, Chicago, IL 60615, USA; admin@projectbrotherhood.net; 5 University of Illinois Cancer Center, 818 S. Wolcott St., Chicago, IL 60612, USA; oseib@uic.edu (O.B.); nutallkimberly@gmail.com (K.N.-T.); 6 Department of Head & Neck Surgery & Communications Sciences, School of Medicine, Duke University, Durham, NC 27710, USA; nosa.peters@duke.edu; 7 Duke Cancer Institute, Duke University, Durham, NC 27705, USA

**Keywords:** black men, oral health, health inequities, oral and pharyngeal cancers, patient–dentist communication

## Abstract

Background: Poor oral and pharyngeal cancer (OPC) survival among Black men is partially due to their limited knowledge about OPCs, which is exacerbated by dentists’ limited training and discomfort in discussing OPC risk factors. The purpose of this study was to assess the attitudes and experiences that Black men have communicating with dentists about OPCs. Methods: To qualitatively assess these attitudes and experiences, a focus group guide and recruitment strategy were developed using a community engagement approach. Data were analyzed using grounded theory. Results: Twenty-three self-identified Black men participated in three focus groups through the Zoom platform (mean age of 46.1 years). Four main themes emerged, which identified that participants: (1) had little knowledge of OPCs; (2) felt that addressing OPC risk among Black men was not a priority for dentists; (3) stressed the importance of dentists acknowledging the complexity of how race and gender affects Black men’s healthcare experiences; and (4) expressed a benefit to receiving information from multiple social networks. Conclusion: The focus groups provided context for how dentists might engage with Black men in discussions about OPC prevention and treatment.

## 1. Introduction

Black men have the lowest 5-year survival from oral and pharyngeal cancers (OPCs) compared to men with other racial/ethnic identities, despite having lower incidence rates [1]. Surveillance, Epidemiology, and End Results data from 2013–2019 reported that Black men’s relative 5-year survival from OPCs was 54.1% compared to 68.2% among other men [2]. Since the early 2000s, there has been a national focus on addressing OPC disparities among Black men. Healthy People, an initiative designed to guide health promotion and disease prevention in the U.S., has set a target to increase the proportion of oral and pharyngeal cancers detected at the earliest stage from 29.5% to 34.2% by 2030 [3]. However, there are few published reports of targeted interventions to reduce Black men’s risk and poor OPC outcomes [4].

The elevated risk of OPCs in Black men, with resultant poor outcomes, are primarily due to a lack of awareness about: OPC risk factors, importance of screening, and disease symptoms [5,6,7]. Reasons why Black men may not seek OPC screening include limited financial resources, fear, lack of symptoms, pride, fatalism, and mistrust of dental providers [4,5,6]. Understudied components of increased OPC risk in Black men specifically are structural inequities, such as limited access to dental care, the lack of racial concordance among dental providers, and the influence of bias and discrimination towards Black men, which can reduce the uptake and quality of their dental care experiences, including patient-centered communication about OPCs [8,9]. When diagnosed with OPCs, it has been shown that Black men have not received the same types of treatment recommendations as other population cohorts [10]. Mitchell and Perry revealed that Black men who lacked health insurance and perceived more discrimination had fewer patient-centered communication interactions than White men [11].

Even when race is not taken into consideration, dentists do not routinely engage in OPC discussions or screening due to structural inequities such as dentists’ lack of training to address their own discomfort, confidence, limited clinical time to discuss OPCs, and a lack of dental insurance reimbursement for OPC screening [12,13]. Another barrier to communication about OPCs is that discussing OPC risk factors of alcohol use, tobacco use, diet and nutrition, and human papillomavirus (HPV) may be uncomfortable for patients and dentists [14,15,16]. For example, a study by Raja and colleagues concluded that dental patients were less comfortable discussing trauma, stress, coping, and living and behavioral patterns than basic demographic information, which has implications for improving dental providers’ understanding of how social determinants may increase patients’ risk for OPCs [17].

Taken together, previous research indicates that OPC-related communication among dentists is poor to begin with [12,13,14,15,16], and health-related communication in general, including OPC-related communication, is even poorer when it involves Black men [6,7,11]—which directly contributes to disparities in OPCs. The complexities of dentists’ and patients’ discomfort warrant an in-depth exploration of how systems and provider communication should be targeted by multilevel anti-racist interventions, which may alleviate inequities in OPC survival. As a preliminary approach for providing context for targeted interventions in improving OPC communication among Black men and dentists, the purpose of this study was to conduct focus groups with Black men in the Chicago area to explore the attitudes and experiences that Black men may have about OPCs and the role of dentists and healthcare systems to reduce OPC burden.

## 2. Materials and Methods

This exploratory, grounded theory qualitative study was approved by the UIC Institutional Review Board (Protocol # 2022-0658) to conduct focus groups with Black men in the Chicago area. This theoretical framework is most commonly used when seeking to discover or construct theory from data, systematically obtained through comparative analysis [18]. A community engagement approach was used to develop a focus group guide based on previous knowledge and experiences of Project Brotherhood, a community-based organization in Chicago, Illinois, with expertise in engaging Black men in cancer research [19,20]. Project Brotherhood also led the study recruitment. Inclusion criteria for the study were self-identified Black men, aged 18 and over, residing in Cook County, Illinois.

Purposive sampling was used to identify eligible study participants, with a target of engaging at least 20 men to achieve saturation and meet study aims. Project Brotherhood led the recruitment effort using their community engagement approach of interacting with Black men in everyday settings to build trusting relationships while promoting wellness and healthy behaviors. Men who were recruited for this study were part of Project Brotherhood’s network of men who had previously participated in sponsored community events, research, and/or other health promotion events. Members of Project Brotherhood’s staff contacted men within their network by means of in-person interactions, text messages, and emails and asked them to participate in this study. Focus group participants were asked to provide written consent prior to participation and agreed to be audio recorded. Prior to the start of each focus group, participants were asked to complete a brief questionnaire asking them of their age, education levels (less than high school, high school diploma or GED, college degree, and graduate degree), time since their last dental visit (less than one year ago, 1 to 3 years ago, and 4 or more years ago), and their comfort levels speaking to dentists about oral cancer (not very comfortable, not comfortable, neutral, comfortable, and very comfortable). Those data were analyzed using SPSS Version 25.0 (Chicago, IL, USA) to calculate mean age, frequencies, and percentage distributions.

Trained research personnel affiliated with Project Brotherhood conducted each of the three focus groups, which were recorded and conducted on the Zoom platform to achieve the following aims: (1) gain a better understanding of knowledge about oral cancer among Black men, (2) identify factors that impact experiences when accessing dental care, and (3) elevate opportunities for increased engagement and awareness of oral cancer. Written field notes were taken by a member of the research team. Audio recordings were transcribed verbatim using an online transcription service. Data were cleaned by a member of the research team by reviewing each transcription and matching it to the recorded audio files to ensure accuracy. Using grounded theory as the selected theoretical framework, several rounds of open coding was used to identify themes. In support of addressing bias, data analysis was conducted by another member of the research team that did not participate in the focus groups or cleaning of transcripts [21]. The cleaned transcripts and field notes were reviewed line-by-line for initial coding and then followed by another round of focused, structural coding that resulted in identifying overarching themes from the responses to each question. Continuous rounds of coding of each document helped to refine themes prior to final analysis and interpretation of focus group findings. Coded data were then grouped into themes using an inductive approach, merging and providing meaning to components or fragments of ideas or experiences. The researcher used Excel spreadsheets to document the frequency of each theme, elevating quotes to support findings. Throughout the analysis process the researcher kept short, descriptive memos to document ideas during preliminary data review and to define codes during coding. Codes and memos were reviewed and discussed by the research team, allowing for modifications and clarifications to the code scheme and definitions. Findings from the final report were shared and formally discussed with the project’s Advisory Committee that included individuals with a range of public health roles, including dentists, dental assistants, community health workers, etc.

## 3. Results

A total of twenty-three men (*N* = 23) participated in three focus groups with a mean age of 46.13 (SD = 13.32). The majority of participants had a minimum education of a college degree (74.9%), had a routine dental checkup less than one year prior to participation (56.5%), and expressed being at least somewhat comfortable discussing oral cancer with their dentists (69.6%). The average duration of the focus groups was 94 min.

Four major themes emerged from the focus group discussions, which are explained below.

Theme #1: Knowledge and perceptions of Black men relating to OPC 

Qualitative study findings highlighted that participants’ knowledge ranged from not knowing anything about OPCs to sharing risk factors that contributed to OPC incidence, such as smoking, chewing tobacco, dip products, and alcohol consumption. Participants who had no knowledge of OPCs expressed the desire to learn more by asking questions of the focus group facilitator in real time. Examples of participant responses supporting these findings included “*no, it’s new to me….hadn’t heard of it*”; and “*[I] haven’t heard a lot about it. I understand that people get it from smoking, or you can catch it from smoking. But other than that, I really don’t know a lot about it*”.

Beliefs about the importance and relevance of OPCs among participants were largely influenced by their personal experiences and limited exposure to OPC promotion that was geared toward Black men. Personal experiences shaping beliefs were described by participants as “*having no loved ones impacted by oral cancer*”; “*only hearing about the risks of oral cancer because of participation in sports*”; and “*this is something that we don’t talk about in the community*”.

Furthermore, participants shared perceptions about how the lack of OPC promotion efforts geared towards Black men contributed to beliefs that OPC was not as important or as relevant as other cancers. Cancer promotion efforts are often linked to increasing conversations and knowledge within the community. Participants stated things such as “*my grandfather had lung cancer…. that’s the closest I’ve come to cancer*”. “*I just want to say the only reason it’s not important to me is because many others said you don’t hear about it as much*”. “*There’s plenty of data out there to support colonoscopies and things like that, but I haven’t seen any data to support Black men, or being disproportionately affected, or even just encouraging black men to go get screened for or to prevent this type of cancer*”.

Despite the lack of exposure and limited knowledge, participants highlighted their interest in wanting to learn more and how to prevent OPC occurrence. When asked “is oral cancer something that is or has been important to you as a Black man?”, participants collectively shared responses highlighting the importance of knowing about all cancers because of the higher rates of incidence and poorer outcomes impacting their communities.

Theme #2: The role of dentistry in cancer prevention and detection practices

Participants questioned the role of dentistry in cancer prevention and detection practices. Prior experiences with dentists largely shaped their attitudes, beliefs, and expectations about the types of activities that should be performed by a dentist. None of the participants had ever experienced a dentist assessing their knowledge about OPCs and/or educating them about OPCs. These experiences seemed to incite skepticism about whether dentists were trained to detect and/or treat OPCs, whether dentists were only concerned about providing services that generate revenue, and whether detection is a part of a dentist’s role. One participant stated that “*dentists look at that as actually your primary care health. They don’t look at oral cancer as part of dentistry….They know about oral cancer, but that’s not their focus*”. Another participant stated that “*…telling you about oral cancer is not making them money. That’s why dentists don’t get into that*”.

These comments reflect the notion that some Black men may not be aware of how much emphasis dentists should place on OPCs, which may affect their personal level of engagement around the topic. For example, there was some support for dentists playing a more formal role in routinely asking about behaviors associated with increased risk for OPCs, including smoking, vaping, and oral sex. Reasons provided included comments such as “*they dropped the ball on this one, this is the dentist’s job to ask these questions*”; “*I would be surprised if they asked because it isn’t widely done, but this should be the norm*”; and “*yes, they should [discuss OPCs] so they can educate the patients about the dangers*”.

Participants shared mixed responses about who should be responsible for providing information to patients about OPCs, based on perceived training of dentists and dental hygienists to detect OPCs. One participant stated that “*I don’t think that a hygienist or anyone like that should really be having that discussion with us*”. Another participant mentioned that “*the hygienists, actually, they are trained to look for oral cancer. Dental assistants are not, so you shouldn’t be having a conversation with them, because they don’t know what they’re looking for*”.

There was some mention of receptionists providing educational materials as a catalyst for prompting the patient and dentist to have a formal discussion during visits. Some participants’ responses discussed no preference for who provided the information because of their interest in having increased engagement about the topic. However, while those individuals were open to having others in addition to the dentist and/or dental hygienist initiate discussions, they stressed that it was the dentist’s role to have a follow up conversation to close the loop. For example, “*the receptionist can give the pamphlet…something to start the conversation, but the discussion should happen with the dentist*”.

Theme #3: Contexts for addressing Black men’s oral health

A key objective for the focus groups was to explore factors that are associated with how Black men perceive their dental care experiences, what seems to work, and what seems to not work. For example, a range of questions were posed to uncover participants’ attitudes about how providers address their concerns and other issues that may be important to them such as race and gender. Several participants stressed the importance of having greater representation of Black dentists in the field—as many of them expressed seeking out Black providers for care. There was mention of feeling more comfortable with Black providers due to a sense of relatability.

As told by one participant, “*relatability between me and my provider is important. I don’t want to feel judged about my behaviors*”. However, participants also shared both favorable and unfavorable experiences with providers despite being the same race. Of importance was having providers that are willing to develop a relationship with patients by taking the time to get to know them and assess their knowledge by asking questions in a non-judgmental, inquisitive manner. This also coincided with participants stressing the importance of chairside manner, including dentists’ body language, word choices, and overall demeanor. Participants expressed wanting to be “*treated by someone that cares and makes you feel comfortable*”. “*When it comes to a doctor, I* [*want*] *somebody that gives a [expletive] about me, or I feel that from them. If I don’t get it the first time, then I don’t go back and I look again*”. Another participant stated that “*I’m really selective about [the] dentist…I come across somebody who shows a sincere concern about my wellbeing and about my health, you know what I mean, I’ll probably [be] more comfortable talking to that person*”. It was also mentioned that “*I think that [body and facial expressions] matter for any situation, especially talking to the doctor because it is the person that you really need to confide in and need to trust what they say is accurate. Body language and facial expressions [are] huge*”. This participant went further and told a story, stating that, in one encounter with a doctor, “*…everything was kind of, I wouldn’t say a joke, but it was kind of like some sort of laughable remark to everything. And it caused me to not see them again because I need to have real conversations and I didn’t need to have someone trying to make light of everything that we talked about*”.

Provider knowledge about Black men’s health was also mentioned as being important. Several participants shared examples regarding the need to seek out providers that possess an understanding of the historical health behaviors of Black men and the behavioral risks associated with Black men’s health. As one participant stated, some dentists “*only pull teeth and don’t help save your teeth, [and don’t] understand the trauma behind what they are doing*”.

Additionally, participants discussed how negative societal perceptions, coupled with stigma about Black men, resulted in inequitable treatment practices, system mistrust, and varied levels of comfort among Black men when engaging the healthcare system. Participants shared sentiments of being regarded as hyper-aggressive and/or intimidating to non-Black individuals before any interactions even take place. One participant stated that “*there’s usually that momentary period, first time visit, that you have to get through the preclusion with other races. Even some of our own sometimes. As soon as they see that you’re Black, you come in, they got to feel you out as much as you’ve got to feel them out. That’s something that most other races don’t have to deal with. So as Black men, that’s a measure that we ourselves have to put out. I mean, just walking down the street, we deal with that. So, you’re walking to a doctor’s office and even if you coming in with the best insurance, whether they know it or not, there’s still that period of time that has to happen for people to get comfortable with each other*”.

Theme #4: Media that support engagement with Black men about OPCs

Participants spoke of the importance of using multiple methods and media to ensure adequate reach within and across communities. As one participant shared, “*my thing is cast the net wide….try every platform, every avenue, or every structure that you possibly can, provided that you have time, resources, and connections*”. Examples of media and methods mentioned included the use of educational flyers, pamphlets, posters, and social media. Providers were encouraged to think of using traditional settings to push out educational materials and messaging like the doctor’s office waiting rooms and non-traditional settings where Black men convene, i.e., barber shops and churches.

Several participants also discussed the role of peer-to-peer learning and sharing among Black men and women that could support building awareness about OPCs in the community. The more these individuals are engaged and learn about OPCs, the more they can serve as messengers to others. Black women were cited as being integral in the promotion of health awareness and health seeking behaviors among Black men by one participant who stated “*I think that you can’t really reach Black men fully without Black women within that as well. You know, everybody listens to their grandma or their mom. They look to them for health information. So I think that’s a super important point*”. In addition, some participants talked about how their participation in the focus group helped to increase their awareness and how having more forums like it could be beneficial. An example of a supporting comment was “*I think that more of these would be positive to spread the word and increase the focus around the awareness of oral cancer. And so for me, yeah, I think it would be positive to grow this in scale just to bring it to more people*”.

## 4. Discussion

Findings from this study aligned with what has been previously reported in the literature about Black men’s lack of knowledge about OPCs, their desire for more information, and dentists’ limited engagement with them about the subject [5,6,7,10,13]. Results also provided insights on Black men’s perspective of the role of dentistry in oral cancer prevention and the overall perception of dentistry that aligned with assertions that have been made regarding dentistry existing as a commercial enterprise rather than prioritizing the health needs of individuals most at risk for dental diseases [22]. Black men’s observation that dentists’ lack of emphasis on helping them reduce their OPC risk is problematic. Dentists’ lack of engagement with Black men around OPCs limits opportunities for prevention, whereas potentially exacerbating Black men’s historic distrust of healthcare providers [23].

Data from this study also emphasized how Black men may perceive power differentials with healthcare providers as threatening or limiting their sense of agency and control in decision-making about their health. Establishing better communication between Black men and dentists may be enhanced with more emphasis on the importance of trust in relationship building, and the effect of perceived judgment that dentists may project onto Black men during dental visits [11,18,23]. The issue of perceived judgment suggests that the association between trust and communication is bidirectional for Black men, possibly explaining why dentists may be uncomfortable and/or reluctant to engage Black men in OPC discussions [12,15,16]. Communication may be enhanced by dentists appearing more interested in relationship building, acknowledging an awareness of how Black men experience racism and negative stereotyping in society, and empowering Black men to play an active role in their care delivery through a person-centered approach.

Possible approaches to OPC communication mentioned in our results have been implemented in colorectal and prostate cancer prevention strategies [24,25,26,27,28]. For example, Black men may benefit from targeted, culturally appropriate messaging because it allows Black men to see themselves represented among high-risk populations. Such messaging may also create comfortable environments where OPC discussions can easily commence. Peer to peer communication has also been lauded as a preferred method of communication among Black men, as it addresses mistrust and values interpersonal relationships, such as those with friends and family members, including women [26,29,30].

One of the significant strengths of this study was how the community engagement approach successfully recruited Black men to participate in focus groups and share their experiences in a trusted environment, which has been cited as a limitation of engaging Black men in research [19]. Men were engaged across a variety of ages and education levels. In addition, a Black man not associated with the dental profession facilitated each focus group, which may have eliminated any pressure among participants to mince their words or withhold their opinions about dentists that may have been unfavorable. A limitation of the study was that the sample consisted of a relatively small cohort of Black men in Chicago, IL. Additionally, the study population may not be representative of local communities. However, the community-engagement methods used in the study helped to determine if the sample population was adequate to meet the research aims and address the needs of the community. Although this limits the generalizability of the study’s findings, the study results provide baseline data needed to help inform future mixed method studies with more participants to more-fully assess factors related to thematic findings in a more generalizable population of Black men. Another limitation was the potential for bias to be incorporated into how data were interpreted. The research team did not record an official audit trail. However, the research team attempted to minimize bias by reviewing coding as well as incorporating field notes into the data analysis process. Future research could expand and diversify the sample size more to determine if Black men of different sociodemographic profiles share similar views. In expanding the study population, the research could also utilize more approaches to improve scientific rigor.

## 5. Conclusions

This study provided a deeper understanding of Black men’s perspectives about communicating with dentists about OPCs, including not receiving knowledge from dentists about OPCs, and the importance of dentists playing an active role in relationship building to establish bidirectional comfort and trust. Dentists can utilize various methods for communicating, such as community outreach and peer-to-peer exchanges. The themes identified in this study will be used to create a culturally aligned OPC communication tool aimed to improve how dentists and Black men engage in discussions about OPC risk and prevention.

## Data Availability

The data presented in this study are available on request from the corresponding author. The data are not publicly available due the authors’ uncertainty about data sharing at the time the manuscript was submitted for publication.

## References

[1] Weatherspoon D.J., Chattopadhyay A., Boroumand S., Garcia I. (2015). Oral cavity and oropharyngeal cancer incidence trends and disparities in the United States: 2000–2010. Cancer Epidemiol..

[2] National Cancer Institute, Surveillance, Epidemiology, and End Results Program SEER Explorer. Oral Cavity and Pharynx. https://seer.cancer.gov/statistics-network/explorer/application.html?site=3&data_type=4&graph_type=5&compareBy=race&chk_race_1=1&chk_race_3=3&series=9&sex=2&age_range=1&stage=101&advopt_precision=1&advopt_show_ci=on&hdn_view=0#resultsRegion0.

[3] Office of Disease Prevention and Health Promotion (ODPHP) nd. Healthy People 2030. Increase the Proportion of Oral and Pharyngeal Cancers Detected at the Earliest State-OH7. https://health.gov/healthypeople/objectives-and-data/browse-objectives/oral-conditions/increase-proportion-oral-and-pharyngeal-cancers-detected-earliest-stage-oh-07.

[4] Satcher D. (2003). Overlooked and underserved: Improving the health of men of color. Am. J. Public Health.

[5] Awojobi O., Scott S.E., Newton T. (2012). Patients’ perceptions of oral cancer screening in dental practice: A cross-sectional study. BMC Oral Health.

[6] Choi Y., Dodd V., Watson J., Tomar S.L., Logan H.L., Edwards H. (2008). Perspectives of African Americans and dentists concerning dentist-patient communication on oral cancer screening. Patient Educ. Couns..

[7] Howell J.L., Shepperd J.A., Logan H. (2013). Barriers to oral cancer screening: A focus group study of rural Black American adults. Psychooncology.

[8] Sabbah W., Gireesh A., Chari M., Delgado-Angulo E.K., Bernabé E. (2019). Racial discrimination and uptake of dental services among American adults. Int. J. Environ. Res. Public Health.

[9] Wright J.T., Vujicic M., Frazier-Bowers S. (2021). Elevating dentistry through diversity. J. Am. Dent. Assoc..

[10] Tomar S.L., Loree M., Logan H. (2004). Racial differences in oral and pharyngeal cancer treatment and survival in Florida. Cancer Causes Control.

[11] Mitchell J.A., Perry R. (2020). Disparities in patient-centered communication for Black and Latino men in the U.S.: Cross-sectional results from the 2010 health and retirement study. PLoS ONE.

[12] Awojobi O., Newton J.T., Scott S.E. (2015). Why don’t dentists talk to patients about oral cancer?. Br. Dent. J..

[13] LeHew C.W., Epstein J.B., Kaste L.M., Choi Y.K. (2010). Assessing oral cancer early detection: Clarifying dentists’ practices. J. Public Health Dent..

[14] Smith P.D., Noorullah K., Iqbal L., Tomar S.L. (2021). Dental students’ comfort discussing nutrition and obesity prevention with parents and caregivers. J. Dent. Educ..

[15] Griner S.B., Thompson E.L., Vamos C.A., Chaturvedi A.K., Vazquez-Otero C., Merrell L.K., Kline N.S., Daley E.M. (2019). Dental opinion leaders’ perspectives on barriers and facilitators to HPV-related prevention. Hum. Vaccin. Immunother..

[16] Parish C.L., Pereyra M.R., Pollack H.A., Cardenas G., Castellon P.C., Abel S.N., Singer R., Metsch L.R. (2015). Screening for substance misuse in the dental care setting: Findings from a nationally representative survey of dentists. Addiction.

[17] Raja S., da Fonseca M., Rabinowitz E.P. (2020). Patient preferences on sharing private information in dental settings. J. Am. Dent. Assoc..

[18] Chun Tie Y., Birks M., Francis K. (2019). Grounded theory research: A design framework for novice researchers. Sage Open Med..

[19] Murray M., Campbell C., Kendall L., Whitt-Glover M.C., Watson K.S. (2019). Perspectives from Project Brotherhood: Facilitating engagement of African American men in research. Prog. Commun. Health Partnersh..

[20] Smith P.D., Murray M., Hoffman L.S., Ester T.V., Kohli R. (2022). Addressing Black men’s oral health through community engaged research and workforce recruitment. J. Public Health Dent..

[21] Poggenpoel M., Myburgh S. (2003). The researcher as research instrument in educational research: A possible threat to trustworthiness?. Education.

[22] Moeller J., Quiñonez C.R. (2020). Dentistry’s social contract is at risk. J. Am. Dent. Assoc..

[23] Hammond W.P. (2010). Psychosocial correlates of medical mistrust among African American men. Am. J. Community Psychol..

[24] Lucas T., Thompson H.S., Blessman J., Dawadi A., Drolet C.E., Hirko K.A., Penner L.A. (2021). Effects of culturally targeted message framing on colorectal cancer screening among African Americans. Health Psychol..

[25] Ward S.H., Lin K., Meyer B., Bass S.B., Parameswaran L., Gordon T.F., Ruzek S.B. (2008). Increasing colorectal cancer screening among African Americans, linking risk perception to interventions targeting patients, communities and clinicians. J. Natl. Med. Assoc..

[26] Walsh-Childers K., Odedina F., Poitier A., Kaninjing E., Taylor G. (2018). Choosing Channels, Sources, and Content for Communicating Prostate Cancer Information to Black Men: A Systematic Review of the Literature. Am. J. Mens. Health.

[27] Woods V.D., Montgomery S.B., Belliard J.C., Ramirez-Johnson J., Wilson C.M. (2004). Culture, black men, and prostate cancer: What is reality?. Cancer Control.

[28] Odedina F.T., Walsh-Childers K., Young M.E., Kaninjing E., Krieger J., Pereira D., Dagne G., Askins N., Fathi P. (2022). Development of a Minority Prostate Cancer Research Digest: Communication Strategy Statement for Black Men. J. Cancer Educ..

[29] Okoro O.N., Nelson C.S., Witherspoon S.P., Witherspoon S.F., Simmons G.E. (2020). Culturally Responsive Health Promotion to Address Health Disparities in African American Men: A Program Impact Evaluation. Am. J. Mens. Health.

[30] Wippold G.M., Frary S.G., Abshire D., Wilson D.K. (2021). Peer-to-peer health promotion interventions among African American men: A scoping review protocol. Syst. Rev..

